# Predictors of Knowledge, Attitude, and Practice (KAP) Towards Family Planning (FP) Among Pregnant Women in Fiji

**DOI:** 10.1007/s10995-023-03618-3

**Published:** 2023-02-13

**Authors:** Mohammed Imtishal, Masoud Mohammadnezhad, Philip Baker, Sabiha Khan

**Affiliations:** 1 School of Public Health and Primary Care, Fiji National University, Suva, Fiji; 2 School of Public Health & Social Work, Queensland University of Technology, Queensland, Australia; 3 Nadi Sub Divisional Hospital, Nadi, Fiji; 4 School of Nursing and Healthcare Leadership, University of Bradford, Bradford, UK; 5 Department of Health Education and Behavioral Sciences, Faculty of Public Health, Mahidol University, Nakhon Pathom, Thailand

**Keywords:** Family planning, Pregnant women, Predictors, Knowledge, Attitude, Practice, Fiji

## Abstract

**Objective:**

This study aimed to determine the predictors of Knowledge, Attitude and Practice (KAP) towards Family Planning (FP) among pregnant Fijian women.

**Methods:**

A cross-sectional study was conducted over two months in 2019 with adult pregnant women attending the Antenatal Clinic (ANC) at Ba Mission Hospital (BMH), Fiji. Data was collected using a self-administrated questionnaire. Statistical analysis included correlation tests and regression analysis in determining predictors of KAP.

**Results:**

240 pregnant women participated in this study with a mean age of 26.02 (± SD = 4.13). The results showed a moderate level of knowledge (mean 14.95, SD ± 3.15), positive attitude (mean 20.56, SD ± 5.68), and good practice (mean 4.97, SD ± 1.73). Linear regression identified that women with more than seven children had a knowledge score of 3.65, lower than null parity (t value = -2.577, p = 0.011). Women aged 20 to 24 had a 6.47 lower attitude score than women aged 18 to 19 (t value = -2.142, p = 0.033). Women in defacto relationships had a 2.12 lower attitude score compared to the married category (t value = -2.128, p = 0.034). Fijian women of Indian descent had a 1.98 lower attitude score than the I Taukei women (t value = -2.639, p = 0.009). Women aged 30–34 had 2.41 lower practice scores than those aged 18–19 (t value = -2.462, p = 0.015).

**Conclusion:**

This study found a medium knowledge of FP among pregnant women. These findings support a recommendation for further research to implement effective strategies.

## Significance

### What is Already Known?

The number of children and spacing interval appears to be influenced by knowledge and contraception usage. The high fertility rate also results from low contraception usage and low knowledge and attitudes toward FP among reproductive-age women.

### What does this Study Add?

Fijian women’s knowledge, attitudes and good practice toward family planning differ from women of other ethnicities in the study. The number of children predicts the attitude towards family planning among Fijian women. Women’s age, marital status, and ethnicity predict attitudes toward family planning. Women’s age is a predictor of practice towards family planning among Fijian women.

## Background

According to the World Health Organization (WHO), “Family Planning (FP) allows individuals and couples to anticipate and attain their desired number of children and the spacing and timing of their births” (Matovu et al., 2017; Wanyenze et al., 2013). FP is achieved through contraceptive methods and the treatment of involuntary infertility (Elizabeth et al., 2002).

The number of children and spacing interval appears to be influenced by knowledge and contraception usage (Wagner et al., 2013). FP has a significant impact on reducing maternal and child mortality. The relevance of FP in any strategy for safe motherhood and child survival is clear (Littleton-Gibbs et al., 2004; Starbird et al., 2016). FP is critical in a woman’s life and is part of their right to choose and control fertility. FP supports the well-being of women and children. Although high fertility rates and rapid population growth affect the economy, it also affects the nation, especially in developing countries, because it has triggered the limitation of resources along with a more significant economic burden (Starbird et al., 2016; Apanga et al., 2015). High fertility increases health risks for mothers and children, leading to poor quality of life and reducing access to education, food, and employment (Askew et al., 2012; Thapa et al., 2018).

Maternal Child Health (MCH) is one of the priority areas for the Fiji Ministry of Health and Medical Services (MoHMS). Fijian population growth through a high fertility rate since 2009 appears to have resulted in increased poverty (Naidu et al., 2017). In addition, the high fertility rate seems to result from low contraception usage and low levels of FP among reproductive-age women (MoHMS. 2016).

Despite significant efforts to increase awareness of FP and contraception availability, assessment of understanding is often lacking (Bearinger et al., 2007). A woman’s age, husband’s education, wealth, spousal communication, and favourable attitude toward contraception are all associated with contraceptive usage (Azmat et al., 2015; Muhindo et al., 2015; Sultan et al., 2002). Pacific Island Countries (PICs) appeared as under-researched; however, Cammock et al. identified Fijian contraception usage average of 45% nationally, but as low as 22% of them are sexually active women (Cammock et al., 2018). The low level of contraception usage is also associated with the low levels of Knowledge, Attitude and Practice (KAP) of women and their partners (Lincoln et al., 2018). The low prevalence of contraception indicates that knowledge about FP is not reflected in behaviour.

Few studies have examined Pacific Islander women’s KAP, usage determinants and perceptions towards FP. As a result, very little is known about what is required to support the adaptation and implementation of evidence-informed public health interventions in Fiji. This study aimed to determine the level of KAP of FP among pregnant women and factors affecting their FP decision in a Fijian community. By understanding the FP decision, this study seeks to address issues like poverty and Malnutrition in Pacific Island Countries and Fiji. In addition, this study’s findings provide an opportunity to review FP policies and improve them to increase family planning knowledge and attitudes and increase contraception usage to improve the FP rate in the country.

## Materials and Methods

### Study Design and Setting

A cross-sectional study was conducted with pregnant women who attended Antenatal Clinic (ANC) in Ba Mission Hospital (BMH), Fiji, from April 15 to June 1 2019. As the only hospital in the Ba Sub-division, BMH receives referrals from two regional health centres. The region has an estimated total population of 57,568, with about 23,000 women of childbearing age.

### Study Population and Sample

Eligible were pregnant women of all ethnic and religious backgrounds, 18 years and over, self–identified as Fijian Ba residents, who attended the BMH antenatal clinic at any given trimester during the data collection period. This study excluded all non-pregnant women who attend ANC and pregnant women unable or unwilling to participate. Two hundred and forty women were invited to participate from an estimated 400 eligible women. In alternative order of presentation, we invited mothers to participate on randomly selected days over a month. On average, 30 women participated each day to recruit the target of 240 mothers. Women completed the survey only once.

### Data Collection Tool

The self-administered questionnaire was in two parts—the first part comprised patient descriptors such as socio-demographics. The second part measured women’s KAP about family planning, subdivided into three sections. Section One, containing 12 questions, inquired regarding family planning knowledge. For each question, there were three possible responses (yes, no and “I don’t know”). Section Two, containing 15 questions, inquired regarding attitudes. For each question, there were three possible responses (agree, disagree and “don’t have any idea”). Finally, Section Three, containing six questions, inquired regarding practice. For each question, there were two possible responses (yes or no).

For each section, questions were scored between 0 and 2, with Total Points for Section One 24 points, Section Two 30 points, and Section Three 6 points. The correct knowledge answers were quantified as: <15 as “Poor” knowledge (Low level), 15–19 as “Moderate” knowledge (Medium level), and > 20 as “Good” knowledge (High level). For attitude, < 18 was considered as “Negative attitude” (Low level), 18–24 as “Neutral” “Medium level and > 25 “Positive attitude” (High level); and for practice, 0–3 was considered as “Bad” practice and 4 to 6 as “Good” practice. Scores less than 60% were classified as Low, 60 to 79% as Moderate, and 80 to 100% as High (Cleland et al., 2011).

To assess face validity, the questionnaire was administered to ten participants who met the study inclusion criteria to determine the extent the questionnaire covered the core concepts. To assess content validity, the questionnaire was provided to content experts (medical and academic staff) who were familiar with the study and worked in family planning. The experts assessed the questionnaire’s content based on the study’s aim and objective. Modifications occurred after assessing their comments.

### Study Procedure

Pregnant women attending the ANC were introduced to the study by the researcher in the ANC room. Women meeting the study’s inclusion criteria were provided with an information sheet in the preferred language, either English, Hindi or Fijian. The researcher or the translators answered questions about the study. After reading the information sheet, those interested and eligible were invited to participate. Signed consent was obtained, and the researcher kept completed forms secured. The study’s information sheet remained with the participants. Consenting participants, using a pen, completed the questionnaire in their preferred language (English, Hindi or Fijian). Those unable to complete the questionnaire independently were offered assistance. The participants were assured they did not need to complete all the questions and could withdraw at any time without penalty. Participants were assured that the study was voluntary and without incentive payment.

Participants were asked to complete the questionnaire on one day but could return it at their next visit or within two weeks. The researcher held the names and contact details of those retaining the survey for subsequent submission. Completed surveys were submitted into a secured box available in all study areas. After two weeks, persons who had not returned the survey received a reminder. We deemed unreturned forms as non-responders.

### Data Analysis

SPSS Version 22 was used for statistical analysis. Demographic continuous variables such as age were expressed as means and standard deviations. For nominal and ordinal data, we described frequency and percentages. The KAP component’s associations with the independent variables were analysed using ANOVA and Multiple linear regression, and the correlation between the dependent variables was analysed using Spearman and Pearson Correlation. All the tests were set at a 5% level of significance.

### Ethical Considerations

The College of Health Research Ethics Committee (CHREC) of Fiji National University (FNU) and the Ministry of Health’s Fiji National Health Research Ethics Review Committee (FNHRERC) approved the study. The study was conducted following relevant guidelines and regulations.

## Results

The study comprised 240 female participants aged 18 to 44 (mean = 26.02, SD = 4.13, and median = 26). Many participants were 25 to 29 years of age (44.2%), and very few were 40 to 44 years of age (0.4%). More than one-third of the participants followed Christianity (38.3%), whilst the remainder followed Islam (31.3%), Hinduism (28.3%) and others 2.1%. Regarding ethnicity, the majority were of FID (58.8%). The remaining I Taukei (39.2%) and other ethnic groups (2.1%). About two-thirds of participants identified as unemployed (60.8%). Most women already had one or more children. More than half (57.1%) had 1 to 3 children, while 2.1% had seven or more children. Regarding the participant’s area of residence, more than half (55.4%) lived in rural settings. Most participants (41.7%) completed secondary education, while 27.9% completed only primary education. 30.4% had completed tertiary-level education. Many participants (26.3%) had a low annual income in the $15,000-$20,000 income bracket. Most participants (81.3%) were married, while 18.8% were in a de facto relationship (Table 1).

**Table 1:** General characteristics of participants (n = 240)

Variables	Categories	Frequency	Percentage
Age (Years)	18–19	4	1.7
	20–24	87	36.3
	25–29	106	44.2
	30–34	35	14.6
	35–39	7	2.9
	40–44	1	0.4
Religion	Christianity	92	38.3
	Hindu	68	28.3
	Islam	75	31.3
	Others	5	2.1
Ethnicity	I-Taukei	94	39.2
	FID	141	58.8
	Others	5	2.1
Employment	Employed	94	39.2
	Unemployed	146	60.8
Number of children	0	52	21.7
	1–3	137	57.1
	4–6	46	19.2
	> 7	5	2.1
Residency	Rural	133	55.4
	Urban	107	44.6
Education	Primary	67	27.9
	Secondary	100	41.7
	Tertiary or higher	73	30.4
Income (n = 94)	< 15,000	7	2.9
	15,000–20,000	63	26.3
	20,000–30,000	18	7.5
	> 30,000	6	2.5
Marital status	Married	195	81.3
	Defacto	45	18.8

Table 2 shows the distribution of the participant’s level of KAP. About one-third of participants (n = 73, 30.4%) had a high level of knowledge towards FP. In contrast, most women (n = 161, 67.1%) had a medium level of knowledge of FP, and very few women (n = 6, 2.5%) had a low level of knowledge. Most pregnant women (56.7%) had a high level of positive attitude towards FP, while about (35.8%) had a positive attitude towards FP. Fewer women (7.5%) showed a negative attitude towards family planning. Regarding practice, the participants showed a low level of FP practice (20.4%), whereas those with good practice in family planning constituted only (191, 79.6%).

**Table 2:** Distribution of responses by the level of KAP

Variables	Levels	Frequency	Percentage
Knowledge	High level (> 20)	73	30.4
	Medium Level (15–19)	161	67.1
	Low level (< 15)	6	2.5
Attitude	Positive attitude (> 25)	136	56.7
	Neutral Attitude (18–24)	86	35.8
	Negative attitude (< 18)	18	7.5
Practice	Good (High Level) Practice (4–6)	191	79.6
	Poor (Low Level) Practice (0–3)	49	20.4

The knowledge score was based on 12 questions, with a maximum possible score of 24. The mean knowledge score was 15.0 (± 3.2), identifying participants with a moderate (medium) level of knowledge towards FP. The attitude score was based on five questions, with a maximum possible score of 30. The mean attitude score was 20.56 (± 5.68), > 20 shows that participants had a positive level of attitudes towards FP. Finally, the practice score was based on six questions, with a maximum possible score of six. The mean practice score was 5.0 (± 1.7), which shows that participants had good practice of FP (Table 3).

**Table 3:** Mean of participants’ answers to KAP questions

Variables	Mean	SD
Knowledge	15.0	± 3.2
Attitude	20.6	± 5.7
Practice	5.0	± 1.7

SD: standard deviation, n = 240

Table 4 presents the mean and SD of the participants’ level of KAP towards FP, which depended on the various demographic variables, including age, religion, ethnicity, annual income, education level, residence, number of children, employment status and marital status. There was a statistically significant association between knowledge and marital status (p = 0.01), with those in the defacto relationships having higher knowledge levels. Those with many children showed the least low level of knowledge (p = 0.001). There was a significant correlation between attitude and age (p = 0.006). With persons in the youngest age group having a positive attitude towards family planning.

**Table 4:** Correlation of demographic characteristics of women with KAP towards FP (n = 240)

Variables	Categories	N	knowledge		Attitude		Practice	
			Mean (SD)	p-value	Mean (SD)	p- value	Mean (SD)	p-value
Age (Years)	18–19	4	14.5 ± 6.6	0.362	27.5 ± 1.9	**0.006**	6.0 ± 0.0	0.074
	20–24	87	15.0 ± 3.3		19.8 ± 6.5		5.1 ± 1.6	
	25–29	106	15.3 ± 3.0		20.6 ± 5.1		5.0 ± 1.8	
	30–34	35	14.2 ± 2.5		20.4 ± 4.7		4.3 ± 1.9	
	> 35	8	13.8 ± 4.1		20.8 ± 4.1		5.5 ± 1.4	
Religion	Christianity	92	14.9 ± 3.1	0.213	20.2 ± 5.7	0.088	5.2 ± 1.6	0.188
	Hindu	68	15.5 ± 3.1		21.5 ± 6.7		4.7 ± 2.0	
	Islam	75	14.4 ± 2.8		18.8 ± 5.1		4.8 ± 1.7	
	Others	5	14.8 ± 7.6		25.2 ± 4.8		6.0 ± 0	
Ethnicity	I-Taukei	94	14.7 ± 3.2	0.218	21.5 ± 5.2	0.063	4.8 ± 1.6	0.417
	FID	141	15.0 ± 3.0		19.8 ± 5.9		5.1 ± 1.7	
	Others	5	17.2 ± 5.2		22.4 ± 6.2		5.0 ± 2.2	
Employment	Employed	94	15.1 ± 3.6	0.538	20.5 ± 5.8	0.899	5.0 ± 1.7	0.607
	Unemployed	146	14.8 ± 2.8		20.6 ± 5.6		4.9 ± 1.8	
Number of children	0	52	15.6 ± 3.7	**0.001**	21.2 ± 6.3	0.797	4.7 ± 2.0	0.398
	1–3	137	14.8 ± 2.6		20.3 ± 5.5		5.1 ± 1.6	
	4–6	46	15.4 ± 3.5		20.5 ± 5.7		4.8 ± 1.9	
	> 7	5	10.7 ± 2.1		21.0 ± 5.1		5.6 ± 0.8	
Residency	Rural	133	15.2 ± 3.0	0.222	20.9 ± 5.7	0.341	4.8 ± 1.9	0.113
	Urban	107	14.7 ± 3.3		20.2 ± 5.7		5.2 ± 1.5	
Education	Primary	67	14.9 ± 3.3	0.977	20.2 ± 5.1	0.776	4.9 ± 1.8	0.618
	Secondary	100	15.0 ± 2.9		20.8 ± 5.9		5.1 ± 1.7	
	Tertiary or higher	73	15.0 ± 3.4		20.5 ± 6.0		4.8 ± 1.7	
Income n = 94	< 15,000	7	15.7 ± 6.4	0.569	20.0 ± 10.6	0.377	4.9 ± 2.0	0.716
	15,000–20,000	63	14.7 ± 3.5		20.1 ± 5.2		5.1 ± 1.7	
	20,000–30,000	18	15.9 ± 2.6		20.9 ± 4.6		4.8 ± 1.8	
	> 30,000	6	15.8 ± 4.1		24.3 ± 8.1		5.7 ± 0.8	
Marital status n = 240	Married	195	14.7 ± 2.9	**0.011**	20.7 ± 5.2	0.382	5.0 ± 1.6	0.355
	Defacto	45	16.0 ± 3.8		19.9 ± 7.4		4.8 ± 2.0	

SD: standard deviation

Table 5 identifies the independent variables collectively that could predict only 4.1% of the total knowledge scores (R^2^ = 0.109, adjusted R^2^ = 0.041). Women with more than seven children had a 3.65 lower knowledge score than those without children (t value = -2.577, p = 0.011).

**Table 5:** Regression analysis of demographic characteristics of women and knowledge of FP

Variables	Categories	Est Coefficient	t	P value	95% CIs	
					Low	Upper
Constant		14.607	6.513	0.001	10.187	19.027
Age (Years)	18–19	ref				
	20–24	1.368	0.811	0.418	-1.957	4.692
	25–29	1.666	0.984	0.326	-1.670	5.002
	30–34	1.070	0.606	0.545	-2.409	4.549
	> 35	0.861	0.413	0.680	-3.244	4.966
Religion	Christianity	ref				
	Hindu	0.532	1.070	0.286	-0.448	1.511
	Islam	-0.291	-0.578	0.564	-1.282	0.701
	Others	-0.177	-0.120	0.905	-3.089	2.734
Ethnicity	I-Taukei	ref				
	FID	0.179	0.428	0.669	-0.647	1.005
	Others	1.777	1.248	0.214	-1.030	4.585
Employment	Employed	-0.726	-1.331	0.185	-1.802	0.349
	Unemployed					
Number of children	0	ref				
	1–3	-0.604	-1.122	0.263	-1.664	0.457
	4–6	0.034	0.046	0.963	-1.406	1.474
	> 7	-3.645	-2.577	**0.011**	-6.433	-0.857
Residency	Rural	-0.615	-1.473	0.142	-1.438	0.208
	Urban					
Education	Primary	ref				
	Secondary	0.365	0.569	0.570	-0.898	1.627
	Tertiary or higher	-0.119	-0.198	0.843	-1.301	1.063
Marital status	Married	ref 0.949	1.705	0.090	-0.148	2.046
	Defacto					

(R^2^ = 0.109, Adj R^2^ = 0.041)

From Table 6, all the independent variables could predict only 6.7% of the total attitude scores (R2 = 0.134, adjusted R^2^ = 0.067). Women in the age category 20–24 years had a 6.47 lower attitude score than those aged 18–19 (t value = -2.142, p = 0.033). Defacto women had a 2.12 lower attitude score compared to the married category (t value = -2.128, p = 0.034). Fijian women of Indian descent had a 1.98 lower attitude score than the I Taukei women category (t value = -2.639, p = 0.009).

**Table 6:** Regression analysis of demographic characteristics of women’s attitudes towards FP

Variables		Est Coefficient	t	P value	95% CIs	
					Low	Upper
Constant		31.723	7.903	0.001	23.812	39.634
Age (Years)	18–19	ref				
	20–24	-6.467	-2.142	**0.033**	-12.418	-0.516
	25–29	-5.277	-1.742	0.083	-11.249	0.695
	30–34	-5.541	-1.753	0.081	-11.768	0.687
	> 35	0.794	0.213	0.832	-6.554	8.142
Religion	Christianity	ref				
	Hindu	1.545	1.736	0.084	− 0.209	3.298
	Islam	-0.307	-0.341	0.733	-2.082	1.467
	Others	4.565	1.726	0.086	-0.646	9.776
Ethnicity	I-Taukei	ref				
	FID	-1.980	-2.639	**0.009**	-3.458	-0.501
	Others	0.822	0.323	0.747	-4.203	5.848
Employment	Employed	ref -0.514	-0.527	0.599	-2.440	1.411
	Unemployed					
Number of children	0	ref				
	1–3	-1.205	-1.251	0.212	-3.103	0.693
	4–6	-1.485	-1.136	0.257	-4.063	1.093
	> 7	-2.285	-0.902	0.368	-7.276	2.705
Residency	Rural	ref -0.713	-0.953	0.341	-2.186	0.761
	Urban					
Education	Primary	ref				
	Secondary	0.929	0.810	0.419	-1.332	3.189
	Tertiary or higher	0.016	0.014	0.988	-2.100	2.131
Marital status	Married	ref	-2.128	**0.034**	-4.084	-0.156
	Defacto	-2.120				

(R^2^ = 0.134, Adj R^2^ = 0.067)

From Table 7, all the independent variables could predict only 4% of the total knowledge scores (R2 = 0.109, adjusted R2 = 0.040). Women aged 30–34 years had a 2.41 lower practice score compared to those with 18–19 years old (t value = -2.462, p = 0.015).

**Table 7:** Regression analysis of demographic characteristics of women with Family Planning practice

Variables		Est Coefficient	T	P value	95% CIs	
					Low	Upper
Constant		6.516	5.242	0.001	4.066	8.965
Age (Years)	18–19	ref				
	20–24	-1.314	-1.405	0.161	-3.157	0.529
	25–29	-1.521	-1.621	0.106	-3.370	0.328
	30–34	-2.409	-2.462	**0.015**	-4.337	-0.481
	> 35	-1.286	-1.114	0.267	-3.561	0.989
Religion	Christianity	ref				
	Hindu	-0.395	-1.435	0.153	-0.938	0.148
	Islam	-0.327	-1.171	0.243	-0.876	0.223
	Others	0.504	0.616	0.539	-1.109	2.118
Ethnicity	I-Taukei	ref				
	FID	0.328	1.414	0.159	-0.129	0.786
	Others	0.269	0.340	0.734	-1.287	1.825
Employment	Employed	ref -0.336	-1.110	0.268	-0.932	0.260
	Unemployed					
Number of children	0	ref				
	1–3	0.435	1.458	0.146	-0.153	1.022
	4–6	0.398	0.982	0.327	-0.401	1.196
	> 7	1.305	1.664	0.098	-0.240	2.850
Residency	Rural	ref 0.377	1.630	0.105	-0.079	0.833
	Urban					
Education	Primary	ref				
	Secondary	0.452	1.272	0.205	-0.248	1.152
	Tertiary or higher	0.018	0.055	0.956	-0.637	0.673
Marital status	Married	ref -0.466	-1.511	0.132	-1.074	0.142
	Defacto					

(R^2^ = 0.109, Adj R^2^ = 0.040)

Table 8 shows the r-value and p-value of the KAP scores of participants for FP. The r-value (-0.082) infers that there was a negative relationship between knowledge and attitude. Likewise, the r value (0.098) shows a weak positive relationship between knowledge and practice. The r value (0.098) denotes a weak positive relationship between attitude and practice. Further, as shown in the table, the relationship between knowledge and attitude (p = 0.2), knowledge and practice (p = 0.170), and attitude and practice (p = 0.129) were not statistically significant.

**Table 8:** Relationship between knowledge, attitudes and practice

Variable	R-value	P value
Knowledge vs. Attitude	-0.082	0.204
Knowledge vs. Practice	0.089	0.170
Attitude vs. Practice	0.098	0.129

## Discussion

This study showed the Fijian participants had moderate knowledge, positive attitudes and good practices towards FP. Regarding their knowledge about FP, the mean score of FP knowledge was 14.95 (± 3.15), categorised as moderate. In other words, women are aware of FP concepts, methods and procedures; however, there is an opportunity to improve their knowledge. The completion rate of the questionnaire was 100%.

From this study, it was noted that 57.5% of pregnant women said that they knew about the different types of contraception available, but the level reported here is much lower than a similar study carried out by Ismail et al. (2014) in Iraqi Kurdistan, identifying almost 97% of women knew about FP. A study conducted in Saudi Arabia had similar findings to the current study 80.6% of the participants knew about FP, and 68.1% correctly defined FP. Many women’s FP knowledge could be increased to improve FP practice (Al-Musa et al., 2019). As shown by Saleh et al. (2018), with of women 95% demonstrating overall general knowledge of FP, high levels of knowledge are achievable.

The analysis shows a significant association between the number of children and women’s knowledge of FP. This association might be valid for the five women having more than seven children, but the observed association could be due to other factors such as religion and ethnicity. Nevertheless, the high precision in the association shows that the number of children is directly associated with the level of knowledge. Conceptually, knowledgeable women may have used FP to control the number of kids. It is widely accepted large family size is directly associated with poverty (Oberta, 2005). However, those who have moderate knowledge may undertake ineffective practice. For example, poorer people often have more children to secure caregivers for themselves during their old age (Okanlawon et al., 2010). For instance, Mansour et al. found good knowledge of contraceptive methods among Saudi women, 31.7% compared to 68.3% with poor knowledge and the parity among 57.5% was less than 5 with a mean value of 4.4 ± 2.9. Further, the Saudi study identified two primary reasons for parity > 3: being religious (belief children are a gift of God) and having high social prestige through a large family (Mansor et al., 2015). This finding is quite pronounced in Abdikadir et al. (2018) which compared university-educated Somali to women with lesser or no education. In their study of 360 women of reproductive age, university-educated females had a mean desired family size of 9.3 children, compared to women with less or no education, with a mean of 10.5 children. In addition, each group had a mean of 4.5 children. The high fertility rate was attributed to a lack of contraception availability and poor knowledge.

However, our study shows a strong association between the number of children and knowledge, which is opposite to what Lincoln et al. (2018) which did not find an association of FP knowledge with the number of children. Furthermore, we have found that lower levels of FP knowledge are associated with more children. This lower level can be attributed to half of the participants being from rural areas with higher unemployment. Also, factors like religious and cultural beliefs, husband disapproval, desire for more children and unavailability of contraception and awareness and lack of education on FP appear to be reasons for having more children. The opposite is seen in younger women who might be employed and have a better educational background and fewer children.

The mean score of participants’ attitudes in this study was 20.6 ± 5.7 (out of a maximum possible of 30) identifying those pregnant women with a positive attitude towards FP. Most of the questions were answered favourably, including the ideal number of children, birth spacing and its usefulness, poverty related to FP, the effect of contraception on health, and the impact of some demographic characteristics on FP. The overall agreement with the core elements of family planning was high (86.77%).

Amongst the possible predictors, we initially expected higher age to be associated with positive attitudes toward FP, perhaps reflecting lived experiences. On the contrary, we found that women in the older age group had a lower altitude than those in the younger age group. We speculate that the significant statistical association might be due to the younger generation having a different approach to life, with most of them career-orientated focus. Thus, preferring fewer children who may choose larger families. Similarly, women in the older age group might not be employed or have other contributing factors to attitude may be present such as cultural or religious beliefs. A similar study by Thapa et al. showed that age had a positive correlation with attitude (Thapa et al., 2018); similarly, Endrias et al. showed a significant effect of age on attitude (Endrias et al., 2017). However, a study by Lincoln et al., (2018) conducted in Suva, Fiji, showed the opposite of no significant relationship between age and attitude. As shown in several settings, age can impact attitude, but the direction of the association may vary.

Marital status also appears associated with attitude, as women in a de facto relationship had a lower attitude towards family than married women. This finding is similar to Kasa et al. (2014) who found married women and a more positive attitude to FP than unmarried women. Nyauchi et al. (2014) also found a similar association. In addition, married women appear to have a more positive attitude toward FP due to the need to limit the number of children (Nyauchi et al., 2014). However, a study conducted in Suva, Fiji, failed to observe this association (Lincoln et al., 2018).

Ethnicity may be associated with poor FP due to various myths and misconceptions about FP. Some ethnic groups do not allow FP or discourage FP use. Resistance to FP is often associated with religion, cultural beliefs and negative attitudes. In refugee camps in Nigeria, Owolabi et al. (2010) found no association between ethnicity and religious attitudes, which Kamruzaman et al. (2015) identified. The choice of contraception methods may be cultural (Rasheed et al., 2015), as methods vary by religion and ethnicity, which contrasts with Lincoln et al. that showed positive attitudes towards FP varied considerably by ethnicity (Lincoln et al., 2018).

FP-related practices among pregnant women in Ba, Fiji, appear to be much better when compared to those reported elsewhere. The older age group had lower practice scores than the younger age group, which may be related to why they were pregnant again at an older age. A possible hypothesis is that older women may be more experienced in practising FP than younger women. It is difficult to interpret the FP practice amongst those 18 to 19 years of age as their current pregnancy may have resulted from failed FP. Adeyemi et al., (2008) showed that women 40 to 49 years of age were four times more likely to use contraception than single women (p < 0.001) (33). This earlier research contrasts with the findings by Ismail et al. (2014) suggested that contraception usage decreases after 40 years of age (19). Osmani et al. (2015) found women preferred not to use FP at an early age as they were newly married and sought to start a family early in the marriage rather than after 40 years of age (34). In contrast, to our findings, Lincoln et al. (2018) found no relationship between age and practice of FP. Our findings suggest younger women appear to use contraception to avoid early pregnancy to enable completion of education and developing careers.

### Limitations

Although, this is the first study conducted in Fiji among pregnant women and assessed their KAP towards FP. This study applied a cross-sectional study and lacks establishment of the temporal relationship between pregnancy and family planning knowledge, attitudes and beliefs. Therefore, causal relationships cannot be determined. The study was a sample from only one regional hospital; the findings may not be generalisable to all pregnant women in Fiji. This study applied a self-administrated questionnaire to collect data and may reflect participants’ views at only one-time point. As shown in earlier research, women might change reporting of their opinions in another setting, such as outside the hospital Antenatal care clinic.

## Conclusion

The study identified clear associations of KAP towards family planning with age, marital status and the number of children. Notably, there was a negative relationship between knowledge and attitudes. In addition, the study showed that knowledge towards FP was moderate. In contrast, attitude and practice were high, meaning that women had adequate knowledge of FP but had a positive attitude and good practice towards FP. These findings suggest opportunities for public health to review current policies and practices that could improve the family planning services provided in sub-divisional hospitals.

## Data Availability

The authors will consider the application by third parties for the original data of this study consistent with the study’s ethics approval.

## List of Abbreviations

ANC: Antenatal Clinic
BMH: Ba Mission Hospital
CHREC: College Health Research Ethics Committee
FP: Family Planning
FID: Fijian of Indian Descent
FNHRERC: Fiji National Health Research and Ethics Review Committee
FNU: Fiji National University
KAP: Knowledge, Attitude, and Practice
MoHMS: Ministry of Health and Medical Services
WHO: World Health Organization.
